# Serological response to nifurtimox in adult patients with chronic Chagas disease: An observational comparative study in Argentina

**DOI:** 10.1371/journal.pntd.0009801

**Published:** 2021-10-04

**Authors:** David Vizcaya, Ulrike Grossmann, Frank Kleinjung, Ruiping Zhang, Kiliana Suzart-Woischnik, Sandra Seu, Teresa Ramirez, Leylen Colmegna, Oscar Ledesma

**Affiliations:** 1 Bayer Pharmaceuticals, Sant Joan Despi, Spain; 2 Bayer AG, Berlin, Germany; 3 Bayer HealthCare, Beijing, China; 4 Centro de Chagas y Patología Regional, Hospital Independencia, Santiago del Estero, Argentina; 5 LAT Research, Buenos Aires, Argentina; University of Texas Southwestern Medical School, UNITED STATES

## Abstract

Nifurtimox is indicated in Chagas disease but determining its effectiveness in chronic disease is hindered by the length of time needed to demonstrate negative serological conversion. We manually reviewed long-term follow-up data from hospital records of patients with chronic Chagas disease (N = 1,497) in Argentina diagnosed during 1967–1980. All patients were aged ≥18 years at diagnosis and were either treated with nifurtimox (n = 968) or received no antitrypanosomal treatment (n = 529). The primary endpoint was negative seroconversion (the “event”), defined as a change from positive to negative in the serological or parasitological laboratory test used at diagnosis. Time to event was from baseline visit to date of endpoint event or censoring. The effectiveness of nifurtimox versus no treatment was estimated with Cox proportional hazard regression using propensity scores with overlap weights to calculate the hazard ratio and 95% confidence interval. The nifurtimox group was younger than the untreated group (mean, 32.4 vs. 40.3 years), with proportionally fewer females (47.9% vs. 60.1%), and proportionally more of the nifurtimox group than the untreated group had clinical signs and symptoms of Chagas disease at diagnosis (28.9% vs. 14.0%). Median maximum daily dose of nifurtimox was 8.0 mg/kg/day (interquartile range [IQR]: 8.0–9.0) and median treatment duration was 44 days (IQR: 1–90). Median time to event was 2.1 years (IQR: 1.0–4.5) for nifurtimox-treated and 2.4 years (IQR: 1.0–4.2) for untreated patients. Accounting for potential confounders, the estimated hazard ratio (95% confidence interval) for negative seroconversion was 2.22 (1.61–3.07) favoring nifurtimox. Variable treatment regimens and follow-up duration, and an uncommonly high rate of spontaneous negative seroconversion, complicate interpretation of this epidemiological study, but with the longest follow-up and largest cohort analyzed to date it lends weight to the benefit of nifurtimox in adults with chronic Chagas disease.

**Trial registration:** The study protocol was registered at ClinicalTrials.gov: NCT03784391.

## Author summary

Nifurtimox has been a treatment for Chagas disease since the 1970s, is a World Health Organization Essential Medicine, and was recently approved to treat pediatric patients in the USA. Chagas disease is typically transmitted to humans via insect bites and is endemic in parts of Latin America. Symptoms may be mild or absent in the early acute phase of disease and about two-thirds of individuals remain largely asymptomatic in the subsequent chronic phase. However, some chronic patients experience severe cardiac or gastrointestinal effects up to 30 years after infection. Chronic Chagas disease is treatable, but positive serology can persist long after the parasite has been cleared. Studies of the effectiveness of nifurtimox in the chronic phase of the disease have historically been limited by small size or short follow-up duration. From 3,633 patients with Chagas disease identified during review of hospital records in Argentina, we analyzed data retrospectively from 1,497 adults diagnosed with chronic Chagas disease over a 14-year period (1967–1980), looking for negative seroconversion among individuals who received nifurtimox or were untreated. The study had limitations that impact interpretation of the findings: treatment regimens and the amount of time patients were followed both varied, and many untreated patients unexpectedly underwent spontaneous negative seroconversion. However, patients treated with nifurtimox were at least twice as likely to be seronegative as untreated patients, providing supporting evidence for the effectiveness of nifurtimox in adults with chronic Chagas disease.

## Introduction

American trypanosomiasis, or Chagas disease, is a zoonosis caused by the protozoan *Trypanosoma cruzi* (*T*. *cruzi*), which is mainly transmitted in endemic countries by bloodsucking insects that carry the parasite [1]. It is estimated that up to 8 million people are infected with *T*. *cruzi* [1,2]. Most are in the endemic regions of Latin America, but emigration to countries like the USA and Spain means that the prevalence of Chagas disease is increasing in non-endemic countries [3,4]. Chagas disease has two clinical phases: acute and chronic. The acute phase lasts for a few weeks or months after infection and is usually either symptom-free or associated with only mild, non-specific symptoms and signs. The chronic phase is characterized by fluctuating parasitemia, but most patients remain asymptomatic for several months or years, during which they are classified as having the indeterminate form of Chagas disease [5]. About two-thirds of infected people will remain in this asymptomatic stage for life. However, among the remainder, serious consequences occur 10 to 30 years after initial infection, with most patients experiencing cardiac and/or digestive tract pathologies [1].

Currently, only two compounds, nifurtimox and benznidazole, are licensed for the treatment of Chagas disease [1,5]. In several South American countries, nifurtimox was approved in the 1970s for the treatment of adult and pediatric patients with Chagas disease, based on clinical studies conducted in the late 1960s [6]. The drug has been successfully used since then and has been included in the World Health Organization (WHO) Model List of Essential Medicines since 1977 [7,8]. Recently, based on new clinical evidence, the US Food and Drug Administration approved the use of nifurtimox to treat pediatric patients with Chagas disease [9,10]. Historically, the chronic form of Chagas disease was neglected as a candidate for antitrypanosomal chemotherapy owing to a scarcity of evidence and underdiagnosis [11,12]. However, evidence from animal and human studies shows that both acute and chronic Chagas disease respond to antitrypanosomal treatment [13–17], and this therapy is now also recommended in clinical guidelines both for adult and pediatric patients with the chronic form of the disease [18]. Most of this research focused on benznidazole, and only a few small studies included nifurtimox in their assessment of antiparasitological effects in chronic disease [19–21].

We wanted to collect more data on the effectiveness of nifurtimox in chronic Chagas disease, but one of the barriers to prospective evaluation is the length of time needed to assess seronegative conversion, which is currently the best indicator of parasite clearance [22]. Data from a retrospective and prospective cohort study deduced that decades of post-intervention follow-up are required to show seronegative conversion in nifurtimox-treated patients [23], which impedes the conduct of well-controlled clinical trials of antitrypanosomal treatments. Retrospective medical chart review offers an alternative, feasible means of obtaining both demographic and long-term clinical data. Thus, we compared the effectiveness of nifurtimox with that of no antitrypanosomal treatment by reviewing all follow-up data from a relatively large, single-center cohort of adult patients in Argentina who were diagnosed with chronic Chagas disease between 1967 and 1980.

## Methods

### Ethics statement

Before the start of the study, documented approval was obtained from the Comité de Ética en Investigación Clínica (the Independent Ethics Committee/Institutional Review Board for the participating center), and the study was conducted according to Good Clinical Practice and as required by local laws, applicable regulations, and participating organizations. Informed consent was not required because all patient data were anonymized. The study protocol was registered at ClinicalTrials.gov: NCT03784391.

### Study design and population

**Fig 1:**
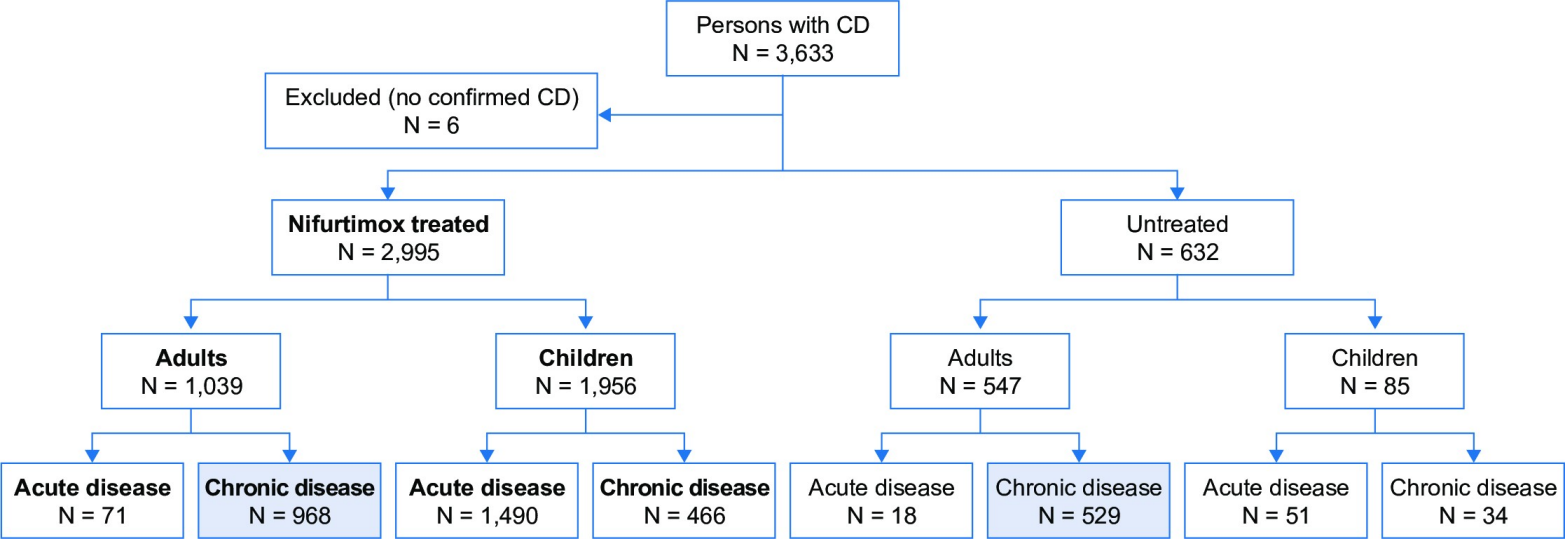
Flowchart of the overall study population. Analysis of the effectiveness of nifurtimox was confined to nifurtimox-treated and untreated adults with chronic Chagas disease (shaded boxes). Adverse events in association with treatment were summarized for all patients who received nifurtimox (bold text). CD, Chagas disease.

We conducted an observational, retrospective study between December 2018 and September 2019 of secondary data obtained from medical records for a cohort of patients with Chagas disease attending the Centro de Chagas y Patología Regional. Centro de Chagas is a large reference center for the diagnosis, treatment, and follow-up of patients with Chagas disease, and is part of Hospital Independencia, Santiago del Estero, Argentina. Clinical and demographic data were collected, as well as information on the evolution of Chagas disease. We included all males and females of any age who had a Chagas disease diagnosis confirmed by a serological (e.g., enzyme-linked immunosorbent assay [ELISA], indirect hemagglutination assay [IHA], indirect immunofluorescence assay [IFA], complement fixation [Machado–Guerreiro][24]), parasitological (e.g., hemoculture, xenodiagnosis), and/or supplementary serological (e.g., immunoblot assay) method in the study period from 1966 to 1980, either before starting antitrypanosomal treatment or during their first antitrypanosomal treatment cycle. The disease stage assigned to each patient (acute, chronic) was that given in the medical records. The study was powered to analyze the effectiveness of nifurtimox in adults (aged ≥18 years) diagnosed with chronic Chagas disease during the study period, who were either untreated or received nifurtimox. During the period covered by this retrospective analysis, default medical practice at Centro de Chagas was not to administer antiparasitic drugs to patients with chronic Chagas disease; however, some physicians elected to offer such treatment, based on their clinical judgment rather than on specific patient clinical criteria or characteristics. As well as examining treatment effectiveness, we summarized adverse events associated with nifurtimox treatment in all patients who were diagnosed with acute or chronic Chagas disease during the study period. Fig 1 summarizes the distribution of the study population.

### Inclusion and exclusion criteria

Participants of the overall study had to be male or female patients aged ≥0 years at diagnosis of Chagas disease confirmed by parasitological and/or serological method(s) before start of antitrypanosomal treatment or during the course of this antitrypanosomal treatment cycle for the first episode recorded. The following exclusion criteria applied to all study participants: participants could not be on treatment with antitrypanosomal agent(s) other than nifurtimox, including experimental investigational products for Chagas disease or antitrypanosomal combination treatment. Likewise, study participants could not have any known evidence of organ manifestation of chronic Chagas disease, e.g., Chagas disease-related cardiomyopathy/heart disease or digestive disease, at time of primary diagnosis because it would be impossible to determine whether individuals with signs of advanced disease had previously received antiparasitic treatment. Of all study participants, only adults with diagnosed chronic Chagas disease during the study period who were untreated or who received nifurtimox were included in the present analysis.

The effectiveness study cohort included 1,497 adults with chronic Chagas disease aged 18 years or more at the time of diagnosis (first patient diagnosed on 28 March 1967, last patient on 23 December 1980), who were either treated with nifurtimox (n = 968) or who did not receive antitrypanosomal treatment (the “untreated” group; n = 529). The cohort reviewed for safety (n = 2,995) included 1,039 adults (including 71 adults with acute disease) and 1,956 children (1,490 with acute disease, 466 with chronic disease) treated with nifurtimox (first patient diagnosed on 8 October 1966, last patient on 30 December 1980).

### Treatment exposure

Information was captured about start of treatment, dose, treatment duration, and interruption of therapy, including reasons and details of any switch to another antitrypanosomal agent. In addition to patients treated as per routine care, adult patients diagnosed with chronic Chagas disease who had participated in previous clinical studies were included in the study; any who had been randomized to placebo were included in the reference population for the period of placebo administration before they switched to an antitrypanosomal agent. Information on concomitant drug treatments at baseline was also collected.

### Outcomes

The primary endpoint was negative seroconversion in chronic Chagas disease. Time to event was the time from baseline visit to the date of the endpoint event. If no event was observed, the time was censored at the last follow-up visit at which the same test method as that used at baseline was administered, or at the visit when an alternative treatment was first administered. Evaluable patients either had an endpoint event or were censored. The primary outcome was defined as a change in laboratory test result (serological or parasitological) from positive to negative measured with the method used in the initial diagnosis. The laboratory methods used for diagnosis are described under “Study design and population.” Changes in applied laboratory test results over time were described for all patients.

### Data collection

Data collected from patient medical records included: demographics, signs and symptoms, results of laboratory testing for Chagas disease, clinical and electrocardiogram (ECG) findings. Furthermore, occurrence of cardiac manifestations in patients with chronic Chagas disease was evaluated by clinical or ECG signs and symptoms, and intake of drugs for the treatment of cardiomyopathy or heart failure; details of the cardiac signs and drug treatments are in S1 Text. Since detailed information about the quality and completeness of the data source was not available when the study was designed, the study protocol provided for a descriptive pre-analysis when data collection was complete for 30% of the patients. This preliminary review of selected variables in the sub-sample informed preparation of the statistical analysis plan. A summary report of this pre-analysis was available, resulting in an amendment to the initial study protocol (according to current international good pharmacoepidemiology practices [25]), namely, the data frequencies indicated that analysis of comparative effectiveness should be confined to adults with chronic disease. The terms “acute,” “latent” or “chronic” were used regarding the clinical phases of Chagas disease in the medical charts; “latent” or “chronic” Chagas disease were both considered to be chronic Chagas disease in this study.

### Statistical analysis

We used descriptive statistics to characterize the nifurtimox and reference cohorts (for continuous variables, mean and standard deviation; for categorical variables, percentage of the corresponding total). Only symptoms occurring in at least five individuals from either the nifurtimox or the untreated group were reported. Between-group comparisons of effectiveness of nifurtimox were calculated using Cox proportional hazard regression analysis. Propensity scores with overlap weights were applied to calculate the hazard ratio (HR) and 95% confidence interval (CI) for the nifurtimox group versus the untreated group. Propensity score adjustment is a standard method to reduce bias in observational settings. The propensity score is the likelihood to be in the treatment group calculated from baseline characteristics. This likelihood is used to weight the impact of the respective patient in the statistical analysis. Overlap weights are a particular type of weighting (and have been accepted by regulatory authorities on several occasions). Thus, the technique mimics randomization to some extent, by reducing confounding bias. Propensity scores were determined by logistic regression, including baseline factors (sex, body weight, age, participation in a nifurtimox clinical trial). Details of the determination of overlap weights based on propensity scores are in S2 Text. A sensitivity analysis was conducted that stratified patients into five equal quintiles based on their propensity score and included test method as a covariate. Kaplan–Meier plots of the cumulative hazards are also provided.

In the analysis stratified by laboratory test method, the model required non-overlapping strata. Therefore, for patients with multiple methods at baseline, the method with the earliest event during follow-up, or with a longer follow-up period without an event, was included in the analysis. If multiple methods had the same follow-up time and the same status, selection was based on the level of representation in each of the respective method groups. Ideally the model would have strata that were equally populated, so to avoid empty strata, the test method representing the group with the fewer patients was the one included for analysis. Secondary endpoints were treatment of cardiomyopathy and/or heart failure, and occurrence of cardiac manifestations as observed in echocardiography, chest X-ray, ECG, 24-hour Holter ECG, or Kuschnir classification referred to as normal/abnormal findings. Only ECG results are reported here because of small sample sizes for the other parameters; analysis of ECG data was exploratory as the study was not powered to detect between-group effects on this parameter. All analyses were conducted using SAS version 9.4 (SAS Institute; Cary, NC, USA).

## Results

### Baseline characteristics

The demographic and clinical characteristics at baseline of adults with chronic Chagas disease are summarized by treatment group in Table 1. The group treated with nifurtimox was younger than the untreated group (mean, 32.4 vs. 40.3 years) and there were proportionally fewer women in the nifurtimox group (47.9%) than in the untreated group (60.1%). Of the 968 nifurtimox-treated patients, 280 (28.9%) had clinical signs and symptoms of Chagas disease at diagnosis, the most frequent being fatigue (10.2%) and headache (8.1%); 25.5% of signs and symptoms in this group were listed as “Other.” Of the 529 untreated patients, 74 (14.0%) had signs and symptoms of Chagas disease at diagnosis, the most frequent being fatigue (2.5%), hepatomegaly (2.3%), and chagasic subcutaneous edema (2.1%); 10.2% of signs and symptoms in this group were listed as “Other.”

**Table 1:** Baseline characteristics of adults with chronic Chagas disease by treatment group.

Characteristic	Nifurtimox (N = 968)	Untreated (N = 529)
Age, mean ± SD, years	32.4 ± 11.1	40.3 ± 13.5
Female, n (%)	464 (47.9)	318 (60.1)
Weight, mean ± SD, kg	68.4 ± 13.7	67.2 ± 13.8
Any symptom, n (%)	280 (28.9)	74 (14.0)
Other	247 (25.5)	54 (10.2)
Fatigue	99 (10.2)	13 (2.4)
Headache	78 (8.1)	9 (1.7)
Malaise	10 (1.0)	3 (0.6)
Subcutaneous edema	8 (0.8)	11 (2.1)
Lymphadenopathy	7 (0.7)	1 (0.2)
Signs of historical portal entry of *T*. *cruzi*^a^	7 (0.7)	1 (0.2)
Hepatomegaly	3 (0.3)	12 (2.3)
Duration of follow-up, median (IQR), days	379.0 (16.5–2549)	46.0 (8.0–1315)

^a^ Sequelae of a chagoma, such as a forunculoid wound, rather than a fresh bite.

IQR, interquartile range; SD, standard deviation; *T*.*cruzi*, *Trypanosoma cruzi*.

Of those treated with nifurtimox, 930 (96.1%) received a known daily dose. The mean maximum daily dose was 8.5 mg/kg/day, median (interquartile range [IQR]) daily dose was 8.0 (8.0–9.0) mg/kg/day, and the overall daily dose range was 3–20 mg/kg/day. Mean nifurtimox treatment duration (n = 967) was 48.3 days (median [IQR], 44.0 [1.0–90.0] days; range, 1–421 days). Concomitant medications were recorded for 144 patients (14.9%) in the nifurtimox group. The most frequently used medications were enalapril (4.2%), prenylamine (3.5%), and amiodarone (2.9%); 2.2% of patients were re-treated with benznidazole. Of the 529 untreated patients, 58 (11.0%) had concomitant medications recorded. The most frequently used medications were prenylamine (3.4%), amiodarone (2.6%), and enalapril (2.3%). One-quarter of the nifurtimox group was followed for at least 7 years (median [IQR] duration of follow-up, 379 [16–2,549] days; range, 1–15,989 days); median (IQR) follow-up duration in the untreated group was 46 (8–1,315) days; range, 1–14,764 days.

The most frequently performed laboratory tests at baseline and during the follow-up period are summarized in Table 2. At follow-up, the frequencies of serology tests (nifurtimox vs. untreated) were: Machado–Guerreiro, 53.1% versus 27.2%; IHA, 35.2% versus 26.5%; and IFA, 22.8% versus 16.6%.

**Table 2:** Frequency of detection methods used with the study participants.

Method	Nifurtimox	Untreated
	(N = 968)	(N = 529)
**Parasitology**	25 (2.6)	12 (2.3)
Parasitological test^a^	4 (0.4)	2 (0.4)
Xenodiagnosis	23 (2.4)	11 (2.1)
**Serology**	562 (58.1)	184 (34.8)
ELISA	101 (10.4)	33 (6.2)
IFA	221 (22.8)	88 (16.6)
IHA	341 (35.2)	140 (26.5)
Latex agglutination test	14 (1.4)	0 (0.0)
Machado–Guerreiro	514 (53.1)	144 (27.2)

^a^ Type of parasitological test was not recorded.

Data are n (%).

ELISA, enzyme-linked immunosorbent assay; IFA, immunofluorescence assay; IHA, indirect hemagglutination assay.

### Effectiveness

Of the 968 nifurtimox-treated adults with chronic Chagas disease, 517 had laboratory results based on the same diagnostic test at both baseline and follow-up. Overall, 54.2% (n = 280) of these patients had negative laboratory test results during the follow-up period. Of the 517 patients, 44 were excluded from the Cox model owing to missing data (sex, body weight, age, or participation in a nifurtimox clinical trial). Of the 473 evaluable patients treated with nifurtimox, 252 (53.3%) had endpoint events and 221 (46.7%) were censored. Among the 529 untreated patients, 165 had data from the same diagnostic test at baseline and follow-up. Overall, 46.1% of untreated patients (n = 76) had negative laboratory test results during the follow-up period. Of these 165 patients, 36 were excluded from the Cox model because of missing data. Of the 129 patients who were evaluable, 61 (47.3%) had endpoint events and 68 (52.7%) were censored. The median time to the first negative result of any laboratory test for nifurtimox-treated patients (n = 517) was 2.1 years (IQR, 1.0–4.5 years; range, 0–33 years) and for untreated patients (n = 165) was 2.4 years (IQR, 1.0–4.2 years; range, 0–29 years). In the Cox model, the estimated HR (95% CI) for time to negative seroconversion was 2.22 (1.61–3.07), favoring nifurtimox over the untreated group.

Stratification of the 473 evaluable patients treated with nifurtimox by test method showed that 360 were tested with the Machado–Guerreiro method, 92 by IHA, and 21 by IFA. Of the 129 evaluable untreated adults, stratification by test method showed 71 patients were tested with the Machado–Guerreiro method, 43 by IHA, and 15 by IFA. Overall, when stratifying qualifying patients (n = 602) into five equal groups according to their propensity scores (each containing approximately 20% of the patients), a balanced distribution of nifurtimox-treated and untreated patients was only observed in the second and third strata (Stratum 2: nifurtimox, 95 [20.1%], untreated, 25 [19.4%]; Stratum 3: 98 [20.7%], 23 [17.8%]; see S1 Table).

**Fig 2:**
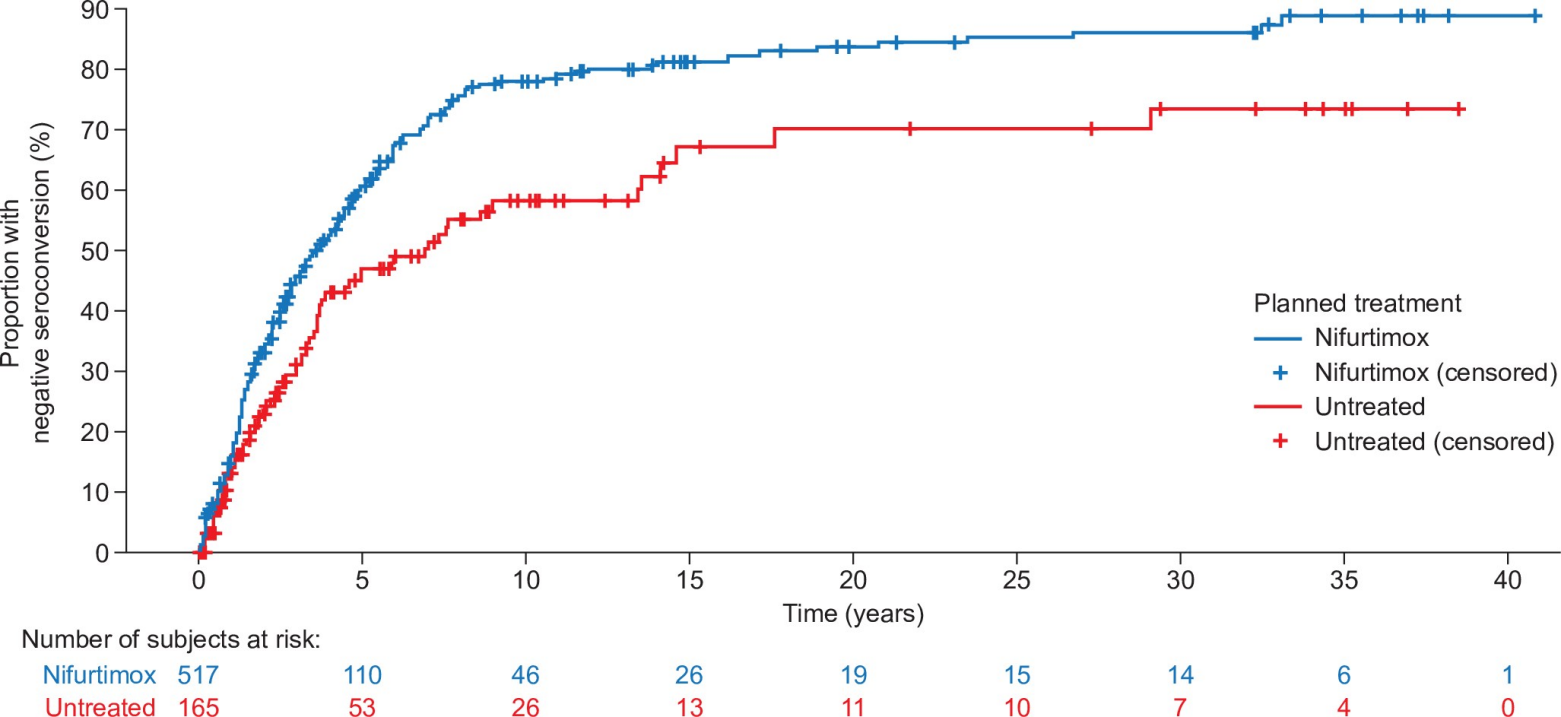
Kaplan–Meier plot for the occurrence of the primary endpoint: negative seroconversion in patients with Chagas disease either treated with nifurtimox or untreated.

Sensitivity analysis by propensity score stratification showed an attenuated effect of nifurtimox on time to negative seroconversion, but the effect was consistent with the main analysis results (HR [95% CI], 1.29 [0.97–1.73]). Kaplan–Meier time-to-event analysis showed that throughout the follow-up period the rate of negative seroconversion was consistently higher among patients treated with nifurtimox than among those not treated (Fig 2).

In addition to negative seroconversion, we evaluated the occurrence of pathological findings on ECG performed during follow-up. The availability of such ECG data decreased during follow-up. At baseline, ECG data were available for approximately 85% of individuals in both the nifurtimox and untreated groups, whereas after 5 years of follow-up, ECG results were available for only 40.7% of the nifurtimox group and 24.4% of the untreated group. In terms of crude association, no relationship between being treated with nifurtimox and having an abnormal ECG was evident in any 5-year follow-up period. Although no clear trend could be established based on crude data, over 35 years of follow-up there was a suggestion that the odds of having an abnormal ECG might have become lower in the nifurtimox group than in the untreated group (Table 3 and S3 Text).

**Table 3:** Occurrence of abnormal ECG during follow-up in patients with chronic Chagas disease and its association with nifurtimox treatment.

Follow-up, years	Nifurtimox		Untreated		Crude OR (95% CI)
	Abnormal/Normal	% Abnormal	Abnormal/Normal	% Abnormal	
Baseline	369/438	45.7	193/247	43.9	1.08 (0.85–1.36)
5	219/175	55.6	66/63	51.2	1.19 (0.8–1.78)
10	71/70	50.4	26/27	49.1	1.05 (0.56–1.98)
15	39/46	45.9	16/14	53.3	0.74 (0.32–1.71)
20	21/37	36.2	9/9	50	0.57 (0.20–1.65)
25	17/25	40.5	7/4	63.6	0.39 (0.10–1.54)
30	20/13	60.6	6/8	42.9	2.05 (0.58–7.29)
35	27/20	57.4	1/0	100	0.68 (0.15–3.03)

CI, confidence interval; ECG, electrocardiogram; OR, odds ratio.

### Safety

In general, all adverse events (AEs) associated with nifurtimox were consistent with its established safety profile, and no new safety concerns were identified. All AEs occurring among the 2,995 patients on nifurtimox who were evaluated in the overall study are listed by frequency in S2 Table. Considering the 968 adults with chronic Chagas disease treated with nifurtimox, 436 (45.0%) had at least one AE. In this group, the most frequently occurring treatment-associated AEs were headache (15.7%), decreased appetite (14.3%), abdominal pain upper (11.2%), nausea (8.4%), dizziness (6.6%), and vomiting (6.0%). Subgroup analyses showed that there was a higher overall incidence of AEs in women (49.6%) than in men (40.9%), and in those diagnosed with Chagas disease in the 1960s (70.0%) and 1970s (46.3%) compared with those diagnosed in the 1980s (23.5%). The same trends in frequency by sex and decade of diagnosis were also observed at the AE level (Table 4).

**Table 4:** Frequency of adverse events among patients with chronic Chagas disease treated with nifurtimox, by sex and by decade of diagnosis.

Event	Sex		Decade of diagnosis		
	Male	Female	1960s	1970s	1980s
	(n = 504)	(n = 464)	(n = 10)	(n = 886)	(n = 68)
**AEs in association with nifurtimox treatment**	206 (40.9%)	230 (49.6%)	7 (70.0%)	410 (46.3%)	16 (23.5%)
**Gastrointestinal disorders**	95 (18.8%)	120 (25.9%)	4 (40.0%)	200 (22.6%)	8 (11.8%)
Abdominal pain, upper	51 (10.1%)	57 (12.3%)	1 (10.0%)	102 (11.5%)	5 (7.4%)
Nausea	33 (6.5%)	48 (10.3%)	2 (20.0%)	74 (8.4%)	4 (5.9%)
Vomiting	21 (4.2%)	37 (8.0%)	1 (10.0%)	54 (6.1%)	2 (2.9%)
**General disorders and administration site conditions**	40 (7.9%)	62 (13.4%)	2 (20.0%)	95 (10.7%)	4 (5.9%)
Asthenia	17 (3.4%)	23 (5.0%)	2 (20.0%)	36 (4.1%)	2 (2.9%)
**Metabolism and nutrition disorders**	61 (12.1%)	78 (16.8%)	4 (40.0%)	131 (14.8%)	2 (2.9%)
Decreased appetite	61 (12.1%)	77 (16.6%)	3 (30.0%)	131 (14.8%)	2 (2.9%)
**Musculoskeletal and connective tissue disorders**	40 (7.9%)	42 (9.1%)	1 (10.0%)	79 (8.9%)	2 (2.9%)
Myalgia	30 (6.0%)	33 (7.1%)	1 (10.0%)	60 (6.8%)	2 (2.9%)
**Nervous system disorders**	90 (17.9%)	114 (24.6%)	1 (10.0%)	194 (21.9%)	7 (10.3%)
Dizziness	25 (5.0%)	39 (8.4%)	1 (10.0%)	60 (6.8%)	3 (4.4%)
Headache	68 (13.5%)	84 (18.1%)	1 (10.0%)	145 (16.4%)	5 (7.4%)
**Psychiatric disorders**	32 (6.3%)	37 (8.0%)	1 (10.0%)	67 (7.6%)	1 (1.5%)
Insomnia	25 (5.0%)	21 (4.5%)	0 (0.0%)	45 (5.1%)	1 (1.5%)
**Skin and subcutaneous tissue disorders**	18 (3.6%)	23 (5.0%)	0 (0.0%)	38 (4.3%)	3 (4.4%)

AE, adverse event.

## Discussion

We reviewed the medical records of male and female patients of any age who had attended the Centro de Chagas in Argentina and been diagnosed with Chagas disease between 1966 and 1980. The study reported here focused on adults with chronic Chagas disease treated with nifurtimox in routine clinical practice, and showed they had a more than twofold higher probability of negative seroconversion than untreated individuals with Chagas disease and similar characteristics. Serological tests to facilitate the diagnosis of Chagas disease have been developed, but there is no definitive test for parasitological cure. To our knowledge, the results reported here were derived from the largest cohort of patients ever used to assess the effectiveness of nifurtimox, or, indeed, of any antitrypanosomal therapy, in patients with chronic Chagas disease, even though only about half of the patients were propensity matched for our effectiveness analysis. Furthermore, the cohort follow-up spans a relatively long period of continuous enrollment since diagnosis, thus contributing to the unique size of the study. Interest in the efficacy of antitrypanosomal treatment for chronic Chagas disease has been growing for many years, and a general consensus on using trypanocidal therapy in adults with chronic disease has been reached, although it is not yet included in clinical guidelines [5,26].

An obstacle to evaluation of the effectiveness of any antitrypanosomal therapy in adults with chronic Chagas disease is the need for extended long-term follow-up, because disease progression is slow and a positive test based on conventional serology can persist long after clearance of the parasite [22,27,28]. Historically, epidemiological studies on the effectiveness of nifurtimox in adults with chronic Chagas disease have usually been undertaken in cohorts of <100 patients with variable follow-up duration [29], and have returned inconsistent results [20,21,23,29,30]. Studies in large patient cohorts have been reported but only followed patients for a relatively short period of time [19]. Our study of >1,000 individuals, followed for several years, addresses the shortcomings of both sample size and follow-up duration, and provides medium- to long-term data that corroborate early evidence for the effectiveness of nifurtimox, and thus for the use of antitrypanosomal treatment, in chronic Chagas disease. In addition, the results might suggest that patients receiving nifurtimox may be less likely than untreated patients to develop cardiac complications, as identified in abnormal ECG tests performed after diagnosis. How ECG findings might be influenced by chronic Chagas disease or by treatment with nifurtimox is not known. At baseline, the most frequent ECG abnormalities were QTc prolongation, low QRS voltage and right bundle branch block, compared with QTc prolongation, right bundle branch block, “other” (repolarization disorder), and low QRS voltage at last ECG. Our findings regarding possible risk reduction in patients receiving nifurtimox should be interpreted with great caution, as the overall number of patients with recorded data was low, particularly beyond 15 years of follow-up. However, perhaps the result provides a foundation for the prospective investigation of whether nifurtimox treatment is indeed associated with such improved outcomes. All the data in our study were collected directly from the patients’ medical charts by trained technicians, thus minimizing any prior selection of patients assigned to each treatment group based on the study protocol. In addition, treatment and disease outcomes were assessed as per routine healthcare management.

Our study has some limitations that must be considered when interpreting the results. Evolution of clinical practice over the study period meant that the criteria for diagnosis of Chagas disease were not uniform across the patient cohort and differed from current WHO recommendations [18]; this shortcoming reflects both the long study period and the observational nature of our investigation. Our data most likely include patients from former Bayer clinical studies with nifurtimox, and patients treated according to clinical standards and guidelines at the time. The clinical studies were performed in the late 1960s and were not randomized. It is also the case that treatment duration could not be precisely determined for many patients who received nifurtimox and that many may have discontinued treatment because of tolerability issues. Median follow-up in the nifurtimox-treated cohort was substantially longer than in the untreated cohort, which might be expected because individuals not being treated would only seek a further consultation if their condition worsened.

It is notable that a substantial proportion of untreated patients (46.1%) underwent negative seroconversion over an extended period of follow-up without having received any antitrypanosomal therapy. Analysis of a prospective cohort of 925 patients with chronic Chagas disease, either untreated or treated with antiparasitic therapy, indicated a spontaneous negative seroconversion rate of approximately 7% in the untreated group, although the criteria for serological conversion were more stringent than in our study [31]. This could imply that the same effect went undetected in the nifurtimox group, lessening the apparent treatment effect, or that the groups had fundamentally different prognoses, which is a possible shortcoming of retrospective observational studies. Default practice during the period of study was not to administer antiparasitic agents to patients with chronic Chagas disease, and physicians who decided to initiate treatment did so based on their own judgment rather than specific clinical criteria. One could speculate that untreated patients had less severe disease (hence the decision not to treat them), perhaps being more able immunologically to counter the parasite than those receiving nifurtimox. Insensitivity of diagnostic tests could have falsely indicated negative seroconversion, although such a factor would probably affect both study groups equally. Whatever the reason, the unexpectedly high rate of spontaneous seroconversion could bias the estimate of effectiveness in the study, but whether both groups or only the untreated group were affected, there remains a significant between-group effect. We address the mixed provenance of study participants in our analysis with propensity scores and subgroup analyses, although a confounding bias is likely in this type of study design. Propensity scores were calculated in a blinded manner and adjusting propensity scores for a range of covariates partly addresses such possible issues. However, it cannot completely allow for other sources of bias such as unmeasured or residual measured confounding. Having adjusted for multiple potential confounding factors, sensitivity analyses still demonstrated an attenuated between-group effect.

A further limitation could be attributable to using various laboratory tests to diagnose Chagas disease with follow-up of serological response over time. The Machado–Guerreiro test was used most frequently in the study, reflecting local standards and test availability during the 1960s and 1970s. Machado–Guerreiro is a complement-fixation diagnostic Chagas test that also had been used, in conjunction with xenodiagnoses, to monitor negative seroconversion following treatment until other, more discriminating, serological tests (e.g., IHA, IFA) became widely available. The sensitivities of both Machado–Guerreiro and xenodiagnoses are relatively low in this regard. After parasite clearance, the Machado–Guerreiro test could remain positive, thus leading to underestimation of clearance rates or to overestimation of time to negative seroconversion. This problem likely happened independently of the treatment received by the patient, thus effecting a non-systematic error in the number of events that would not affect the estimation of effects. In addition, there could be changes in laboratory testing standards during the observation period. This limitation is inherent to long-term cohort studies, but it is unlikely that it affected the two treatment groups differently. Finally, although reflecting actual practice, the quality of documentation could vary among health practitioners even within the same hospital. Missing data due to unrecoverable or unrecorded information, difficulties interpreting text (e.g., jargon, acronyms), and variability in the quality of information recorded by medical professionals are obstacles encountered when extracting data from medical charts, and these may have influenced the validity of our study. We anticipated that this might cause a problem, so to confirm that the data quality and completeness of the variables collected were of an appropriate standard, we conducted a quality check when data from 30% of all included participants had been entered into the database, then adjusted the protocol based on the findings of this quality check.

Seropositivity can persist long after clearance of the parasite, so negative seroconversion in Chagas disease is the best evidence currently available that the parasite infection may have been overcome, even though it is not directly indicative of cure. Indeed, cardiac complications associated with chronic Chagas disease have been reported in patients shown to have undergone negative seroconversion after benznidazole treatment [32]. Notwithstanding the uncertainty of the relationship between negative seroconversion and parasitological cure, achieving negative seroconversion with antiparasitic treatment is intrinsically desirable in certain scenarios, for example among chronically infected women of childbearing potential in whom it can reduce or even ablate vertical transmission of parasite to the unborn child [33].

These results should be considered together with the available body of evidence to inform both clinical guidelines and healthcare practitioners about managing these patients. Indeed, future analyses of this kind could support public health initiatives and best clinical practice in chronic Chagas disease by trying to identify the timing and type of diagnostic test during follow-up that is clinically most informative in terms of parasite clearance, and to characterize the relationship between treatment and long-term outcomes such as cardiomyopathy. Observational research is not the preferred option to establish the efficacy of a drug, but acknowledging this, and in a scenario where randomized clinical trials are not feasible, our well-designed, large epidemiological study provides evidence for the benefit of nifurtimox in chronic Chagas disease.

## Supporting information

S1 Text Clinical signs and drug treatments evaluated as evidence of cardiac manifestations in patients with Chagas disease. (DOCX)

S2 Text Cox proportional hazards regression: determination of propensity scores and overlap weights based on propensity scores. (DOCX)

S3 Text Regression analysis of the association between treatment with nifurtimox and worsening of ECG findings. (DOCX)

S1 Table Number of patients by propensity score strata and test method (adults with chronic Chagas disease). (DOCX)

S2 Table Adverse events (AE) associated with nifurtimox, by patient group, and by system organ class (≥1% in any group) and preferred term (>1 patient in any group). (DOCX)
