# Supplementary material for: Serological response to nifurtimox in adult patients with chronic Chagas disease: An observational comparative study in Argentina
Source: PLoS Negl Trop Dis. 2021 Oct 4;15(10):e0009801. doi: 10.1371/journal.pntd.0009801 (PMC8489720; doi:10.1371/journal.pntd.0009801)
Supplement: S1 Text — (DOCX) [file pntd.0009801.s001.docx]

**S1 Text. Clinical signs and drug treatments evaluated as evidence of cardiac manifestations in patients with Chagas disease.**

| **Clinical and electrocardiographic signs and symptoms** | **Drugs for treatment of cardiomyopathy or heart failure** |
| --- | --- |
| - left ventricular dilation and dysfunction - cardiomegaly - dilated congestive cardiomyopathy - congestive heart failure - thromboembolism/thromboembolic events (e.g., stroke) - arteriosclerosis - apical aneurysm - sudden cardiac death - right bundle branch block - left anterior fascicular block - ventricular arrhythmias - ST-T changes - abnormal Q waves - low voltage of QRS - various degrees of AV block - sick sinus syndrome - low QRS voltage | - beta-blockers - diuretics - digitalis glycosides - hydralazine nitrate - angiotensin-converting enzyme inhibitors - angiotensin II receptor blockers - antiarrhythmics - calcium channel blockers - aldosterone blockers - anticoagulants corticosteroids |
