# Supplementary material for: Serological response to nifurtimox in adult patients with chronic Chagas disease: An observational comparative study in Argentina
Source: PLoS Negl Trop Dis. 2021 Oct 4;15(10):e0009801. doi: 10.1371/journal.pntd.0009801 (PMC8489720; doi:10.1371/journal.pntd.0009801)
Supplement: S2 Text — (DOCX) [file pntd.0009801.s002.docx]

**S2 Text.** **Cox proportional hazards regression: determination of propensity scores and overlap weights based on propensity scores.**

Propensity scores will be determined using a logistic regression. The baseline factors will include sex, body weight, age, and participation of a clinical trial with nifurtimox.

SAS codes for estimating propensity scores for each patient:

PROC LOGISTIC DATA = adsl;

CLASS trtmnt nftm sex;

MODEL trtmnt (EVENT = nftmx) = nftm sex weight age;

BY test;

OUTPUT OUT = propsc PREDICTED = pscore;

RUN;

Note:

(1) The CAPITALS are the SAS key words,

(2) “adsl” is the subject level analysis dataset, which includes all the parameters for analysis (including patient identification, time to event parameters) of the specific analysis set.
Six parameters (trtmnt, nftm, sex, weight, age, and test) are used for running this logistic regression.

- trtmnt: a binary variable for treatment, “nftmx” for nifurtimox, and “untrt” for untreated
- nftm: a binary variable for participation of a clinical trial, yes or no
- sex: a binary variable for sex, male or female
- weight: a continuous variable for body weight at baseline
- age: a continuous variable for age at baseline
- test: a categorical variable for laboratory test method.

(3) Any missing data will result in a missing propensity score for the patient, which means the patient will be excluded from further analysis

(4) “propsc” is the output dataset from the logistic regression, which includes all the parameters in the dataset “adsl,” plus a new parameter “pscore,” which is the estimated probabilities of being treated with nifurtimox (i.e. propensity scores).

The logistic model might not converge because of limited patient numbers and/or lack of variability in the baseline characteristics. In such a scenario, SAS will output the results based on the last iteration and issue a warning message. Owing to the exploratory nature of the analysis, the process will move on in such cases.

Overlap weights will be determined accordingly as follows:

DATA propsc;

SET propsc;

IF trtmnt = nftmx THEN ow = pscore;

IF trtmnt = untrt THEN ow = 1 - pscore;

RUN;
