# Supplementary material for: Serological response to nifurtimox in adult patients with chronic Chagas disease: An observational comparative study in Argentina
Source: PLoS Negl Trop Dis. 2021 Oct 4;15(10):e0009801. doi: 10.1371/journal.pntd.0009801 (PMC8489720; doi:10.1371/journal.pntd.0009801)
Supplement: S3 Text — (DOCX) [file pntd.0009801.s003.docx]

**S3 Text. Regression analysis of the association between treatment with nifurtimox and worsening of ECG findings.**

Method

We ran a log-binomial regression model for repeated measures to estimate the association between treatment with nifurtimox and a worsening of ECG findings. We considered ECG data as a binary variable (normal ECG or abnormal ECG) both at baseline and at the last ECG performed during the patient’s observation period, and the model was adjusted by age at baseline and sex. Relative risk was calculated using GENMOD in SAS 9.4.

Result

Relative risk (95% confidence interval) of abnormal ECG (nifurtimox vs. untreated): 0.90 (0.81–0.99); *p* = 0.025.

Interpretation

Adjusting for sex and age, the analysis suggests that patients treated with nifurtimox are less likely to develop an abnormal ECG than untreated patients, but other confounding factors that may influence the result cannot be discounted.
