# Supplementary material for: Serological response to nifurtimox in adult patients with chronic Chagas disease: An observational comparative study in Argentina
Source: PLoS Negl Trop Dis. 2021 Oct 4;15(10):e0009801. doi: 10.1371/journal.pntd.0009801 (PMC8489720; doi:10.1371/journal.pntd.0009801)
Supplement: S1 Table — (DOCX) [file pntd.0009801.s004.docx]

**S1 Table.** **Number of patients by propensity score strata and test method (adults with chronic Chagas disease).**

| **Stratum** | **Treatment group** | **IFA** | **IHA** | **Machado–Guerreiro** | **Overall** |
| --- | --- | --- | --- | --- | --- |
| 1 | Nifurtimox | 5/21 (23.8) | 39/92 (42.4) | 21/360 (5.8) | 65/473 (13.7) |
|  | Untreated | 12/15 (80.0) | 33/43 (76.7) | 11/71 (15.5) | 56/129 (43.4) |
| 2 | Nifurtimox | 4/21 (19.0) | 28/92 (30.4) | 63/360 (17.5) | 95/473 (20.1) |
|  | Untreated | 2/15 (11.6) | 5/43 (11.6) | 18/71 (25.4) | 25/129 (19.4) |
| 3 | Nifurtimox | 3/21 (14.3) | 19/92 (20.7) | 76/360 (21.1) | 98/473 (20.7) |
|  | Untreated | 0/15 (0) | 4/43 (9.3) | 19/71 (26.8) | 23/129 (17.8) |
| 4 | Nifurtimox | 3/21 (14.3) | 5/92 (5.4) | 96/360 (26.7) | 104/473 (22.0) |
|  | Untreated | 1/15 (6.7) | 1/43 (2.3) | 14/71 (19.7) | 16/129 (12.4) |
| 5 | Nifurtimox | 6/21 (28.6) | 1/92 (1.1) | 104/360 (28.9) | 111/473 (23.5) |
|  | Untreated | 0/15 (0) | 0/43 (0) | 9/71 (12.7) | 9/129 (7.0) |

Data are n (%).

Propensity scores were determined using a logistic regression including baseline factors: sex, body weight, age, and participation in a clinical trial of nifurtimox. Nifurtimox, N = 968; untreated, N = 529.

Stratum 1: propensity score ≤20th percentile

Stratum 2: 20th percentile < propensity score ≤40th percentile

Stratum 3: 40th percentile < propensity score ≤60th percentile

Stratum 4: 60th percentile < propensity score ≤80th percentile

Stratum 5: 80th percentile < propensity score.

IFA, indirect immunofluorescence assay; IHA, indirect hemagglutination assay; Machado–Guerreiro, complement fixation assay.
