# Supplementary material for: Serological response to nifurtimox in adult patients with chronic Chagas disease: An observational comparative study in Argentina
Source: PLoS Negl Trop Dis. 2021 Oct 4;15(10):e0009801. doi: 10.1371/journal.pntd.0009801 (PMC8489720; doi:10.1371/journal.pntd.0009801)
Supplement: S2 Table — (DOCX) [file pntd.0009801.s005.docx]

**S2 Table.** **Adverse events (AE) associated with nifurtimox, by patient group, and by system organ class (≥1% in any group) and preferred term (>1 patient in any group).**

| **Patient group**  Data are n (%) | **Acute Chagas disease** | | **Chronic Chagas disease** | |
| --- | --- | --- | --- | --- |
|  | **Children**  **(N = 1490)** | **Adults**  **(N = 71)** | **Children**  **(N = 466)** | **Adults**  **(N = 968)** |
| Patients with at least one AE | 675 (45.3) | 39 (54.9) | 151 (32.4) | 436 (45.0) |
| Patients with at least one serious AE^a^ | 8 (0.5)^b^ | – | 1 (0.2)^c^ | – |
| **Primary system organ class**  **Preferred term** | | | | |
| Ear and labyrinth disorders  Tinnitus  Vertigo | 1 (0.1)  – 1 (0.1) | 1 (1.4)  1 (1.4) 1 (1.4) | 2 (0.4)  1 (0.2) 1 (0.2) | 8 (0.8)  4 (0.4) 3 (0.3) |
| Gastrointestinal disorders  Abdominal pain  Abdominal pain, upper  Colitis  Diarrhea  Dyspepsia  Nausea  Vomiting | 255 (17.1)  3 (0.2) 30 (2.0) 2 (0.1) 117 (7.9) 10 (0.7) 54 (3.6) 130 (8.7) | 23 (32.4)  – 13 (18.3) – 1 (1.4) 3 (4.2) 6 (8.5) 8 (11.3) | 68 (14.6)  3 (0.6) 27 (5.8) – 11 (2.4) 3 (0.6) 13 (2.8) 35 (7.5) | 215 (22.2)  7 (0.7) 108 (11.2) – 17 (1.8) 20 (2.1) 81 (8.4) 58 (6.0) |
| General disorders and administration site conditions  Asthenia  Crying  Drug intolerance  Fatigue  Malaise  Pyrexia | 90 (6.0)  3 (0.2) 3 (0.2) 7 (0.5) 8 (0.5) 2 (0.1) 70 (4.7) | 8 (11.3)  3 (4.2) – 2 (2.8) – – 3 (4.2) | 27 (5.8)  8 (1.7) – 4 (0.9) 1 (0.2) 2 (0.4) 13 (2.8) | 102 (10.5)  40 (4.1) – 14 (1.4) 15 (1.5) 13 (1.3) 30 (3.1) |
| Hepatobiliary disorders  Biliary colic | – | 1 (1.4)  – | –  – | 6 (0.6)  4 (0.4) |
| Investigations  Weight decreased | 7 (0.5)  6 (0.4) | –  – | 1 (0.2)  1 (0.2) | 12 (1.2)  12 (1.2) |
| Metabolism and nutrition disorders  Decreased appetite | 409 (27.4)  409 (27.4) | 20 (28.2)  20 (28.2) | 73 (15.7)  73 (15.7) | 139 (14.4)  138 (14.3) |
| Musculoskeletal and connective tissue disorders  Arthralgia  Bone pain  Myalgia  Neck pain | 19 (1.3)  6 (0.4) – 12 (0.8) – | 4 (5.6)  2 (2.8) – 2 (2.8) – | 7 (1.5)  – – 6 (1.3) – | 82 (8.5)  11 (1.1) 5 (0.5) 63 (6.5) 2 (0.2) |
| Nervous system disorders  Amnesia  Balance disorder  Dizziness  Headache  Paresthesia  Paresis  Seizure  Somnolence  Syncope  Tremor | 95 (6.4)  2 (0.1) 2 (0.1) 18 (1.2) 52 (3.5) 2 (0.1) – 20 (1.3) 13 (0.9) 2 (0.1) 1 (0.1) | 15 (21.1)  1 (1.4) – 4 (5.6) 11 (15.5) 2 (2.8) 1 (1.4) – 2 (2.8) – – | 52 (11.2)  2 (0.4) 1 (0.2) 9 (1.9) 39 (8.4) 3 (0.6) 1 (0.2) 1 (0.2) 3 (0.6) 1 (0.2) 1 (0.2) | 204 (21.1)  11 (1.1) 1 (0.1) 64 (6.6) 152 (15.7) 12 (1.2) 3 (0.3) – 10 (1.0) – 7 (0.7) |
| Psychiatric disorders  Agitation  Anxiety  Disorientation  Insomnia  Irritability  Nervousness  Sleep disorder | 103 (6.9)  3 (0.2) 8 (0.5) 1 (0.1) 18 (1.2) 48 (3.2) 39 (2.6) 2 (0.1) | 9 (12.7)  – 4 (5.6) 2 (2.8) 5 (7.0) 1 (1,4) 2 (2.8) – | 13 (2.8)  2 (0.4) – – 4 (0.9) – 7 (1.5) – | 69 (7.1)  4 (0.4) 5 (0.5) 1 (0.1) 46 (4.8) 3 (0.3) 24 (2.5) – |
| Renal and urinary disorders | – | 1 (1.4) | – | – |
| Skin and subcutaneous tissue disorders  Dermatitis  Dermatitis, allergic  Prurigo  Pruritus  Rash  Rash erythematous  Rash macular  Rash maculo-papular  Rash morbilliform  Rash papular  Urticaria | 82 (5.5)  3 (0.2) 9 (0.6) 9 (0.6) 2 (0.1) 36 (2.4) 2 (0.1) 2 (0.1) 5 (0.3) 3 (0.2) 2 (0.1) 5 (0.3) | 6 (8.5)  1 (1.4) – – – 3 (4.2) – – – 1 (1.4) – – | 14 (3.0)  4 (0.9) 1 (0.2) 1 (0.2) 1 (0.2) 7 (1.5) – – – – – – | 41 (4.2)  10 (1.0) 6 (0.6) – 2 (0.2) 14 (1.4) – – – 1 (0.1) – 8 (0.8) |

^a^For more than 50% of AEs occurring among patients receiving nifurtimox, there was no record of whether the event was serious.

^b^Six infants/toddlers (diarrhea, 3; vomiting, 1; rash morbilliform, 1; seizures, 1) and two children (seizures, 2)

^c^One child (toxicity to various agents [“drug intoxication”])

AEs associated with nifurtimox were coded using MedDRA version 22.1.
